# Managing generative-AI health information resilience: human resource capabilities and multilevel public health governance

**DOI:** 10.3389/fpubh.2026.1941787

**Published:** 2026-10-22

**Authors:** Abdelrehim Awad, Mohamed Abbas El-Naggar, Dina Helmy Al Nashily, Hiyam Abdulrahim, Rohit Bansal, Adel Ghonim

**Affiliations:** 1 Department of Business Administration, College of Business, University of Bisha, Bisha, Saudi Arabia; 2 Department of General Subjects, University of Business and Technology, Jeddah, Saudi Arabia; 3 Faculty of Engineering, Chemical Engineering Department, Alexandria University, Alexandria, Egypt; 4 Faculty of Commerce, Department of Business Administration, Al-Azhar University, Cairo, Egypt; 5 Department of Economics, College of Business Administration, Princess Nourah bint Abdulrahman University, Riyadh, Saudi Arabia; 6 Rockford College, Rockdale, NSW, Australia

**Keywords:** AI governance, human resources, conversational generative artificial intelligence, digital public health, health equity, health information resilience, health literacy, organizational resilience

## Abstract

The use of Conversational Generative Artificial Intelligence (GenAI) is growing as a way to interact with health information, especially for the public. This theory-based conceptual synthesis suggests a multilevel governance approach to reducing the likelihood for generated information to become unsafe action – GenAI health information resilience (GenAI-HIR). Its contribution is immediately usable and practical — deploying organizations and public-health actors and public-health actors can use the consequence-based control tiers, actor-specific safeguards, and domain-level audit anchors, and the proposed competency configuration still needs to be validated and is a research agenda for the future. Five conceptually distinct functions at the individual and workforce levels describe at which point protection may contribute to the decision pathway, but are not necessarily assumed to be empirically separable: recognize, interrogate, verify, contextualize, and escalate. Organizational capacity is limited to controls particular to created health information such as authorized sources, attainable human routing, incident monitoring and corrective action. Platform and regulatory conditions are constraints, safety, access, calibrated trust, and equity, outcomes. The framework was created using an expert guided purposive synthesis of the 62 sources that were retained, not a systematic review process. It distinguishes between an explanatory risk model and a normative control architecture and categorizes outputs with respect to type of output, severity, urgency, reversibility, context dependence, mitigation feasibility, and remaining risk. Falsification criteria are now included in six propositions. The candidate self-report indicators are kept as material for the cognitive interviewing only and must be correlated with behavioral vignettes before use for psychometric purposes. GenAI-HIR is therefore not a validated scale, certification standard or replacement for accessible professional care; it is a model for competency that can be falsified in future research and a governance structure that can be audited for current implementation.

## 1 Introduction

The shift from document retrieval (based on document ranking) to conversational (synthesized, translated, and seemingly customized) responses has been enabled by generative AI in the health-information-seeking domain. This change is important to public health as responses may influence how symptoms are understood, how to prevent them, medication choices, institutional rapport, and decisions to seek care. Errors can be especially convincing because of conversational systems' ability to alleviate geographic, temporal, linguistic, and informational barriers, but also because of the apparent fluency, fast talking, empathy cues, and confident personalization. Risks of reported instances include unsupported claims, avoidable red flags, fake references, biased performance, inadequate source disclosure and using outdated guidance (1–19). Although existing scholarship on health literacy, digital health literacy, AI literacy, misinformation-resistance, and epistemic-vigilance is relevant to some aspects of the problem, it does not on its own distribute responsibility for source governance, accessible escalation, or incident correction among users, health organizations, platforms, and regulators. Health-system resilience literature builds in the ability to absorb, adapt to and learn from disruption (20, 21). GenAI-HIR puts that logic into practice with a laser focus on generated health information: ensure access to useful information; detect failures before they manifest as harmful action, contain them, and learn from them.

The article does not aim at claiming that a new psychometric construct has been established, but rather that an actionable multilevel governance framework has been provided. Readers can audit current deployments using the risk tiers and actor responsibilities and consider the five-function competency configuration and its incremental prediction as a secondary empirical research agenda. The evidentiary and escalation requirements of an educational explanation and a directive medication recommendation should not be the same. In this regard, the article (1) introduces the notion of a consequence-sensitive control architecture, (2) tries to identify conceptually different protective functions without assuming empirical separability, (3) separates the individual, organizational, platform and population level, (4) distinguishes explanatory mechanisms from normative safeguards, and (5) specifies propositions, falsification criteria and candidate indicators for future testing.

## 2 Conceptual approach and auditable evidence base

### 2.1 Methodological anchor

This study is a theory informed, conceptual synthesis based on Rodgers' questions for conceptual analyses of evolution (22) and the model, borderline and contrary cases outlined by Walker and Avant (23). It looks at antecedents, attributes, consequences, related concepts, empirical referents, and contextual variation; it is not intended to perform a Rodgers concept analysis or a systematic review. Theory synthesis and negative-case reasoning have been incorporated to render the proposed boundaries and failure points inspectable. The resulting system is open to empirical revision.

### 2.2 Scope, unit of analysis, and terminology

Analytic target is a safe conversation decision process with people accessing health information using conversational GenAI. The four levels are: (i) individual and workforce functions, (ii) organizational GenAI-HIR capacity, (iii) platform and regulatory conditions, and (iv) population outcomes. This multilevel governance configuration is referred to as generative-AI health information resilience (GenAI-HIR). The five functions represent potential protection points for the individual and for the workforce and are not necessarily validated competencies. Organizational capacity is more limited than general responsible-AI governance; it involves controls related to outputs of information generated by the AI relating to approved and existing sources, disclosure of epistemic status, human routing which is readily available, incident capture, corrective action, and subgroup monitoring. However, general governance arrangements continue to be important prerequisites. Safety, calibrated trust, care seeking, access and equity are outcomes, not defining attributes. Strong infrastructure will help to a degree to keep users with low competencies safe, but low competencies will not make up for the lack of verification or care. This architecture thus does not facilitate substituting user judgment for safe systems.

### 2.3 Evidence identification and purposive corpus

The evidence base was curated using purposive selection (with expert advice) and updated on 22 September 2026. It was not a systematic search nor presented with a screening denominator. Discovery and bibliographic verification were done using PubMed/MEDLINE, Google Scholar, Crossref and OpenAlex; targeted retrieval of empirical, theoretical and policy sources were done on the publishers' platforms as well as the official repositories of World Health Organization (WHO), United Nations Educational, Scientific and Cultural Organization (UNESCO), National Institute of Standards and Technology (NIST) and the European Union. Search queries combined the terms “generative artificial intelligence” or “large language model” or “chatbot” with health information, public health, health literacy, AI literacy, misinformation, epistemic vigilance, automation bias, trust, equity, disability, implementation, governance, resilience, measurement, and scale development. In the absence of English-language sources, foundational sources published prior to 2000 were retained as needed. A source was retained if it was used to provide at least one of the following analytic functions: provided a definition of an adjacent construct, explained a risk mechanism, documented health-information performance or user response, specified a governance or accessibility control, or guided operationalization. The total number of sources in the final corpus is 62. Selection was iterative and purposive, no retrospective PRISMA-style count was made. Source clusters, analytic functions and framework decisions are reported in Appendix A.

### 2.4 Concept-development and checking procedure

1. Specify the focal failure: a consequential health decision is shaped by dynamically generated, persuasive, and incompletely contextualized information.
2. Decompose adjacent constructs by object, level, mechanism, and observable behavior; map their contributions against the proposed competencies.
3. Map evidence-supported risk mechanisms from exposure to decision process and outcome, distinguishing empirical evidence, theoretical inference, and illustrative examples.
4. Apply the necessity test: remove one candidate element and specify the safety function that becomes unprotected. Merge candidates that do not add a distinct function.
5. Apply negative-case and level-consistency checks, then formulate propositions whose predictors, moderators or mediators, outcomes, levels, and temporal order do not overlap.
6. Translate the final configuration into preliminary items and audit indicators, while explicitly reserving psychometric claims for future validation.

The audit fields were source, concept, level, mechanism, candidate function, decision, and justification. The authors resolved interpretive differences by consensus. Three boundary decisions were central. First, confidence calibration was treated as an outcome to reduce criterion contamination. Second, equity was treated as a design constraint and outcome criterion rather than an individual competency. Third, organizational learning was defined as an incident-to-correction process within organizational capacity. The necessity test was used only as a conceptual check: it identifies what safety function would be unprotected if a candidate were removed, but it does not demonstrate empirical separability.

### 2.5 Limits of inference and research integrity

The framework is based on conceptual and practical inferences, not prevalence claims, diagnostic claims, causal claims, or psychometric claims. Normative recommendations and hypothesized relationships are in the form, “should,” “may,” or “proposed.” There are no participant(s), primary data or unpublished empirical findings reported. The language editing, consistency in structure and formatting of documents were helped during revision using generative AI. All authors made conceptual decisions, checked the references against publisher records or DOIs, reviewed all revisions, and are fully responsible for the manuscript.

## 3 Theoretical derivation, necessity, and construct boundaries

### 3.1 From theories to functions

The five individual and workforce functions are Recognize, Interrogate, Verify, Contextualize, and Escalate. They are conceptually distinguishable protection points, not empirically proven dimensions. Inoculation theory informs recognition of misleading cues; epistemic vigilance informs examination of source and message; health and digital health literacy inform appraisal and application; AI literacy informs awareness of model status and limitations; human–automation research informs independent checking and escalation; and implementation science explains how controls become reliable organizational practice (24–42). Table 1 presents this theory-to-function derivation and a conceptual necessity argument. If future task-based data show that adjacent functions cannot be differentiated—for example, a collapsed-domain model fits as well as or better than a separated model, indicators lack discriminant performance, or domains do not show different criterion relationships—the functions should be merged or reported as a non-compensatory profile rather than preserved by definition.

**Table 1:** Theory-to-function derivation and conceptual necessity test.

Function	Primary theoretical source	Derived mechanism	Conceptual necessity argument: failure if omitted
Recognize	AI literacy; inoculation (30–37)	Identify generated status, persuasive cues, and task limits before trust is formed.	Fluent output may be mistaken for retrieved or professional advice.
Interrogate	Epistemic vigilance (38)	Probe source competence, benevolence, assumptions, uncertainty, and missing context.	The initial framing and omissions remain unexamined.
Verify	Health/digital health literacy; epistemic vigilance (24–29, 38)	Compare consequential claims with current, independent, decision-relevant evidence.	Self-critique or repeated prompting can reproduce the same unsupported claim.
Contextualize	Health literacy; equity-oriented public health (2, 24–29, 51–53)	Assess clinical, cultural, economic, linguistic, and service applicability.	Factually correct general guidance may still be infeasible or unsafe.
Escalate	Automation-bias and safety-boundary research (4–6, 41, 42)	Transfer decisions to qualified human care when stakes or uncertainty exceed safe self-management.	Automation may delay time-sensitive care or inappropriate self-treatment.
Organizational learning process	Implementation science; risk governance (4–6, 39, 40, 43, 62)	Capture incidents and near misses, analyze causes, implement corrective actions, and monitor recurrence within the broader organizational capacity.	The same failure can recur even when an individual user learns.

The necessity column is a conceptual thought experiment, not evidence that the functions are empirically separable. Future task-based studies must test whether adjacent functions provide distinct prediction.

### 3.2 Conceptual distinctiveness and incremental prediction

GenAI-HIR does not assert that any of the parts and pieces of GenAI-HIR are not available in previous scholarship. It claims novelty for a proposed coordination of functions that takes into account their consequences, with explicit human escalation and organization feedback, under dynamically generated health information. Hence, the distinctiveness is considered to be a function of configuration, level separation and incremental prediction, but not relabeling. Table 2 shows the overlap with neighboring constructs, the health-information specific modification, and the empirical finding that is needed for incremental value. NIST AI RMF offers general govern–map–measure–manage functions (43), WHO guidance offers health-specific ethics, oversight, transparency, risk assessment and post deployment monitoring (4, 5) and CFIR offers implementation determinants (39). Organizational GenAI-HIR isn't a replacement for these frameworks, but rather a distillation of them down to a smaller source-to-decision process that involves health-information provenance, accessible human routing, red-flag handling and recurrence monitoring.

**Table 2:** Capability-by-construct comparison and incremental-value test.

Element	Coverage in adjacent constructs	GenAI-HIR modification	Incremental prediction to test
Recognize	AI literacy addresses capabilities, limits, and algorithmic awareness (30–33).	Links model-status recognition to health-risk classification and action thresholds.	Predicts lower misplaced confidence in fluent, high-stakes errors beyond AI-knowledge scores.
Interrogate	Epistemic vigilance evaluates message and source reliability (38).	Applies vigilance iteratively to changing conversational frames and missing clinical context.	Predicts detection of omitted red flags across dialogue turns beyond generalized skepticism.
Verify	Health/digital health literacy includes appraisal and source evaluation (24–29).	Requires independent, current, jurisdiction- and decision-matched confirmation proportional to stakes.	Predicts selection of higher-quality sources and correction of fabricated citations beyond DHLI scores.
Contextualize	Health literacy addresses application; equity scholarship addresses structural barriers (2, 24–29, 51–53).	Separates surface personalization from clinically and locally valid applicability.	Predicts rejection or adaptation of infeasible advice beyond health-literacy comprehension.
Escalate	Health navigation and automation research partly address referral and over-reliance (27–29, 41, 42).	Defines consequence-sensitive boundaries for leaving the AI channel and reaching human care.	Predicts timely, proportionate care seeking beyond factual knowledge and institutional trust.
Organizational learning process	Implementation and risk frameworks include monitoring and adaptation (4–6, 39, 40, 43, 62).	Specifies the incident-to-correction cycle within, but not equivalent to, organizational GenAI-HIR capacity.	Predicts fewer recurrent failure modes after controlling for general governance maturity and baseline incident burden.
Organizational GenAI-HIR capacity	NIST AI RMF, WHO LMM guidance, and CFIR address general governance, health-AI ethics, and implementation determinants (4, 5, 39, 43).	Links those general functions to generated health-information provenance, accessible human routing, red-flag handling, incident correction, and subgroup monitoring.	Predicts safer routing and fewer repeated health-information failures after controlling for general AI-governance maturity and implementation climate.

### 3.3 Multilevel architecture and cross-level configurations

Table 3 distinguishes the unit, indicators, and role assigned to each level. Four cross-level configurations follow. The intended resilient configuration combines strong competencies with strong infrastructure. Strong competencies with weak infrastructure leave residual risk because authoritative verification and accessible escalation may be unavailable. Limited competencies with strong infrastructure may receive partial protection through defaults, use boundaries, source controls, and human routing. Limited competencies with weak infrastructure form the highest-risk configuration. These configurations permit tests of cross-level interactions without assuming a single undifferentiated construct.

**Table 3:** Units of analysis in the GenAI-HIR configuration.

Level	Construct at that level	Illustrative indicators	Role in the framework
Individual/public user	Five GenAI-HIR competencies	Scenario recognition, quality of interrogation, independent verification, contextual fit, proportionate escalation	Proximal protective competencies
Health workforce	Role-specific five competencies plus documentation	Verification against guidance, communication repair, escalation and documentation quality	Professional protective competencies
Organization	Organizational GenAI-HIR capacity (governance, resources, controls, monitoring, and learning process)	Governance, source control, escalation access, incident response, recurrence reduction	Broader antecedent/moderator; organizational learning is one temporal process within it
Platform/regulator	Enabling and constraining conditions	Provenance, uncertainty design, auditability, reporting duties, enforceable boundaries	Contextual antecedents/moderators
Population	Outcomes	Harm, delay, trust calibration, access, subgroup disparities, repeated incidents	Consequences; not construct items

### 3.4 Model, borderline, and contrary cases

Table 4 uses model, borderline, and contrary cases to test whether the proposed boundaries produce analytically different implications.

**Table 4:** Cases used to test conceptual boundaries.

Case	Description	Analytic implication
Model case	A user recognizes an AI answer as provisional, notices missing medication history, verifies the claim with an official source, and follows a location-specific pharmacist route. The organization records the near miss and corrects the retrieval source.	All five competencies are present; organizational capacity supplies routing, while the learning process converts the near miss into corrective control change.
Borderline case	A digitally skilled clinician detects uncertainty and verifies evidence but cannot provide a usable referral route because the service is unavailable in the patient's language.	Individual/workforce competence is high, but enabling infrastructure and equity performance are weak.
Contrary case: over-reliance	A user accepts confident symptom reassurance, does not check red flags, and delays urgent care.	Absence of recognition, verification, and escalation produces foreseeable risk.
Contrary case: excessive skepticism	A user rejects a correct urgent-care recommendation solely because it came from AI and delays care despite authoritative confirmation.	Resilience requires calibrated trust, not maximal distrust; confidence calibration is an outcome.

## 4 Risk mechanisms in conversational GenAI health information

### 4.1 Persuasive fluency, anthropomorphism, and authority substitution

In situations where evidence is scant, human language, responsiveness, and empathy cues, as well as confident structure can communicate credibility. Earlier studies have shown that anthropomorphism (14, 17, 44–47) and information quality (14, 48) are related to trust and willingness to use health information produced by AI, with prior experience and uncertainty reduction also having been found to be related (14, 17, 44, 46, 47). Previous automation research also indicates that interface and task conditions can lead to over-reliance (41, 42), however, this work precedes the advent of conversational LLMs and serves as a conceptual link rather than direct evidence of the impact of LLMs. The proposed mechanism is authority substitution – communicative quality is considered as evidence of competence. It needs to be directly tested for the magnitude and the distribution of its subgroups.

### 4.2 Hallucination, omission, and unstable reproducibility

Large language models are susceptible to inventing references, creating false causal statements, and failing to provide or misinform instructions, contraindications, red-flagged symptoms and uncertainty (12–19). Emergency-care assessments have identified incomplete advices, faulty source reporting, and sometimes unsafe recommendations, despite fluent presentation (18). Retrieval grounding with high quality sources can mitigate hallucination (49, 50) however, direct evaluations indicate compromises in completeness, readability, or response behavior; and retrieval grounding alone does not guarantee relevant retrieval, recency, jurisdictionality, or accurate synthesis. Consequential claims must be verified separately from the model's own self-critique, however.

### 4.3 False personalization and context mismatch

Conversational systems can repeat back information that users provide and seem individualized, but do not include examination results, thorough history, local resources, disability needs, pregnancy status, medication interactions, or financial limitations. General information can as a result be correct, but not necessarily safe or viable for a specific individual. User studies indicate that users are interested in convenience, accessibility and personalization but also need health professionals to validate them (45–47). This mechanism refers to the relationship between information quality and structural access: information to access specialist care is not protective if there is no structural access available (2, 51–56).

### 4.4 Bias, language inequality, and privacy

Average performance does not necessarily mean that it is sufficient for all subgroups. Algorithmic inequities, unequal access to digital services, language-dependency, and disability-related barriers can lead to unequal distribution of benefits and errors (2, 51–58). Arabic answers were less accurate than English answers for one in three matched cirrhosis questions in an Arabic – English comparison (58) which supports the notion that English language performance cannot be automatically generalized. There is a lack of evidence relating specifically to disabled users of consumer LLM health tools, but this does not mean that they are equivalent. Conversational systems also encourage the sharing of sensitive health information. Platforms and organizations are obligated to minimize data, have easily accessible privacy notices, and have secure human escalation; these are not tests of user competence (4, 5, 43, 59, 60).

### 4.5 Automation bias, delay, and cumulative interaction risk

Previous automation research demonstrates that decision aids lead to omission errors, over-reliance and complacency (41, 42). Trust, planned use, and ongoing use regardless of source quality and correctness are now documented as LLM-specific studies (18, 19, 45–47). When users look for reassurance, hold on to a false premise, or get locked into a first explanation, risk may be cumulative, as the dialogue progresses. It is then possible to copy, translate and re-distribute claims without attribution. The cumulative mechanism is still largely theoretical, so that evaluation needs to focus on whole interaction tracks, corrections and downstream action and not only answer accuracy (16, 17, 34–38).

## 5 Definition and operational content of GenAI-HIR

GenAI-HIR is a provisional multilevel governance mechanism designed to maintain a safe decision-making process if health information is created conversationally. At the individual and workforce level, it outlines five distinct functions that are conceptually different from each other: First, recognize the generated status and stakes; Second, interrogate assumptions, uncertainty, and missing context; Third, verify consequential claims independently; Fourth, contextualize guidance to circumstances with clinical, cultural, economic, linguistic, disability, and service considerations; and Fifth, escalate beyond the AI channel when urgency, potential harm, or uncertainty cannot be safely managed. Within this bounded system property, the Organizational GenAI-HIR capacity is what enables these functions to be actionable through health-information source control, accessible human routing, use boundaries, incident monitoring, resources and accountability. Within that capacity is the incident-to-correction sequence, which is organizational learning. Platform and institutional conditions are enablers, safety, calibrated trust, access and equity outcomes. The observable behaviors and boundaries are combined in Tables 1, 2, respectively, and the previous Table 5 is omitted in order to not repeat the same functions.

**Table 5:** Output type, consequence dimensions, residual risk, and proposed minimum controls.

Output type	Dimension rule	Illustrative tier	Minimum control
Informational	Low severity, not time-sensitive, reversible, and minimally context-dependent	Low residual risk after basic disclosure	AI disclosure, response date, and authoritative source where available
Navigational	Accuracy depends on location, timing, service availability, and accessibility	Low–moderate residual risk	Current jurisdiction-specific route, accessible alternatives, and service-date verification
Interpretive	Applies information to a person; missing context can materially change meaning	Moderate–high residual risk	Missing-context prompts, explicit uncertainty, independent verification, and qualified review
Directive	Recommends action where severity, urgency, or irreversibility may be high	High residual risk, or unsupported for autonomous output	Validated scope, restricted current sources, safety trigger, and immediate human or emergency routing
Any type with red flags	A severe or time-critical dimension requires urgent control unless a tested mitigation reduces residual risk	Normally high residual risk	Actionable accessible escalation; a generic disclaimer is insufficient

WHO, NIST, EU, and disability-access guidance support the general duties of disclosure, oversight, source governance, monitoring, and accessibility (4, 5, 43, 56, 60). The output taxonomy, residual-risk combination rule, and proposed minimums are the authors' synthesis and require empirical and normative validation.

## 6 Separate explanatory and normative models

The explanatory model is given in Figure 1: Exposure characteristics shape risk mechanisms influencing decision processes and outcomes. This pathway can be interrupted by individual functions and controls within the organization. There are two feedback loops that are specifically identified: incident learning informs control and competency, and subgroup monitoring informs access and equity safeguards. The arrows are tentative causal relationships, not necessarily causal effects.

**Figure 1:**
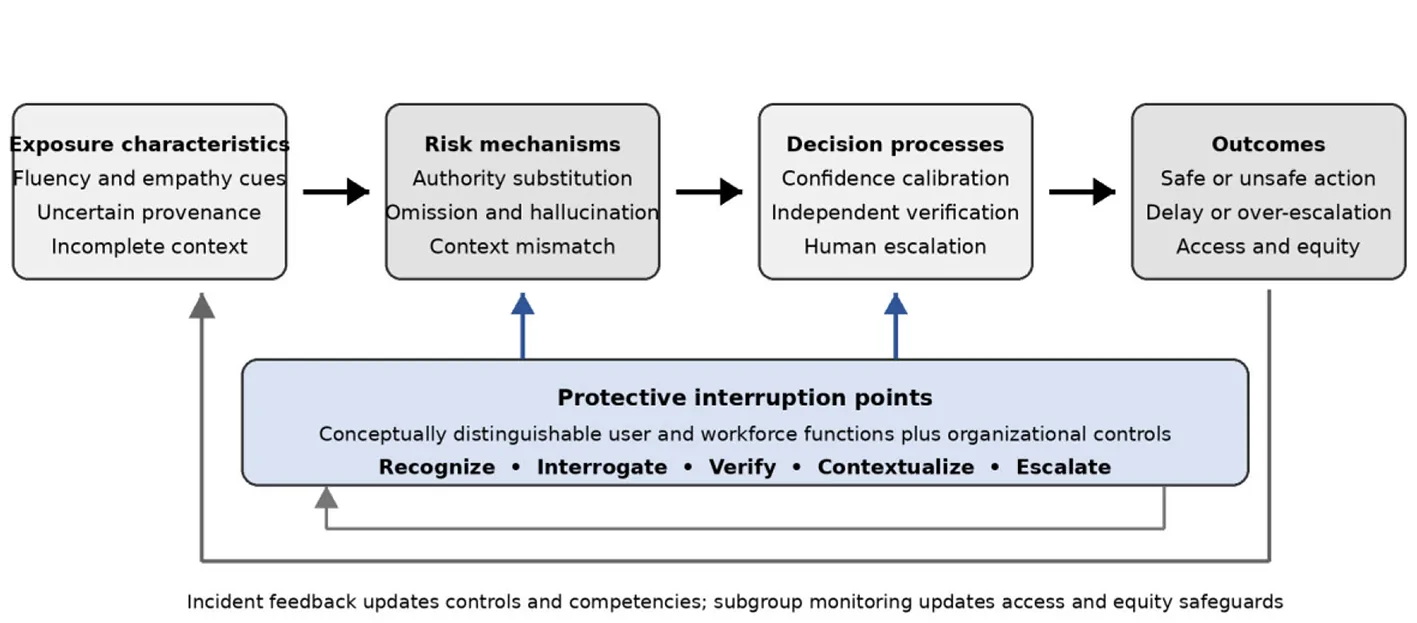
Explanatory risk model for conversational GenAI health information. Solid arrows represent the proposed exposure-to-outcome pathway; protective interruption points and two incident-driven feedback routes are shown separately. Source: Authors' own elaboration.

Figure 2 presents the normative control architecture as a stage-by-actor matrix. It makes visible which controls belong before exposure, during generation, before action, and after action, and which actors can exercise them. Responsibility follows control over risk: users and professionals exercise judgment, while organizations, public-health agencies, platforms, and regulators provide source, interface, escalation, monitoring, and accountability conditions.

**Figure 2:**
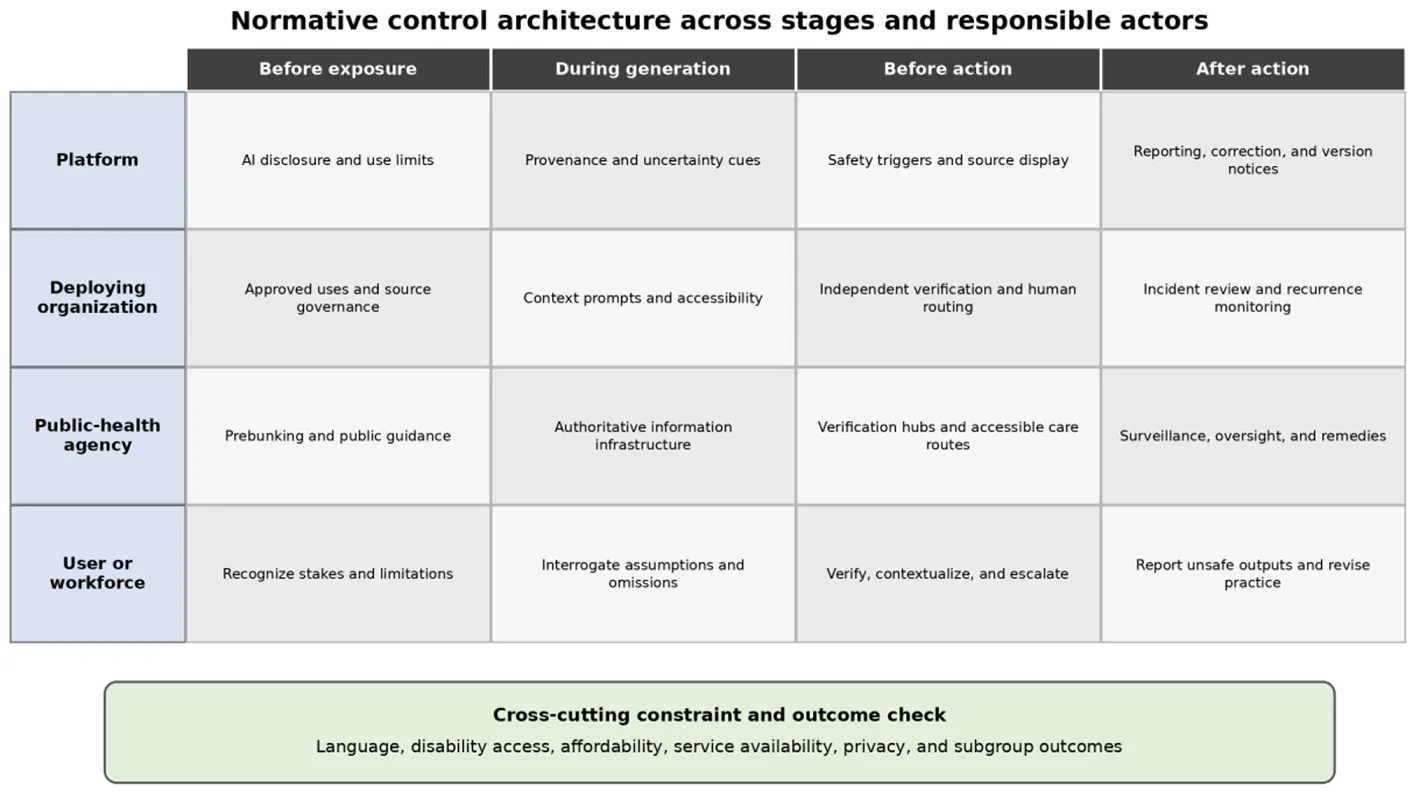
Normative control architecture for conversational GenAI health information. The matrix maps four control stages to platforms, deploying organizations, public-health agencies, and users or the workforce; equity and accessibility apply across all cells. Source: Authors' own elaboration.

### 6.1 Integrated risk classification

The first classification is not a risk score, it is output type. The suggested method is: (1) categorize the response as informational, navigational, interpretive, or directive; (2) rate the severity, urgency, reversibility, context dependence, and access need for different types of users; (3) determine possible safeguards; and (4) apply controls based on the remaining risk after safeguards. Weakness or handicap needs to be followed up with individualized accessibility and routing supports, not with automatic exclusion or automatic access to the highest level. While a maximum rule can be used mechanically, a single severe and time-critical dimension can justify high controls, but one that reduces residual risk is not. The proposed control minimums are shown in Table 5. Disclosure, human oversight, risk management, source governance, monitoring and accessibility principles are based on WHO, NIST, EU and disability guidance (4, 5, 43, 56, 60) while the four types of output, combination rule and operational thresholds are the author's suggestions and are yet to be validated. “Unsupported” indicates that an autonomous directive output should not be deployed if validated scope, current sources, tested safety triggers and immediate human routing are not available.

## 7 Propositions for empirical testing

For each proposition, the predictor, moderator and/or mediator, outcome, level, temporal order, and result that would count against the claim are explained. Interface effects examined are more closely related but different; P2 examines whether the information provided in the interface influences the association of previously acquired competencies with subsequent verification; P6 examines whether the information provided in the interface has a direct causal effect on reliance. The falsification criteria are summarized in Table 6.

**Table 6:** Proposition tests and explicit falsification criteria.

Proposition	Core test	Result supporting the claim	Result that would count against the claim
P1	Incremental prediction across mixed accurate and inaccurate scenarios	The validated configuration reduces confidence–accuracy error and unsafe action beyond adjacent literacies.	No incremental prediction, worse calibration, or benefit confined to an outcome already embedded in the measure.
P2	Competency × provenance or uncertainty-cue interaction	Cues strengthen the competency–verification association.	The interaction is null, reversed, or reproduced equally by decorative noninformative cues.
P3	Mediated and moderated pathway from interrogation to care selection	Red-flag detection mediates the effect and escalation competence strengthens detection-to-action translation.	Interrogation does not predict detection, detection does not predict action, or escalation adds no translation benefit.
P4	Access as a cross-level constraint	Protective associations attenuate when verification or care is inaccessible, with identifiable subgroup differences.	Access has no moderating effect, or the direction is consistently opposite across adequately powered groups.
P5	Multisite longitudinal incident recurrence	Corrective-action completion mediates fewer recurrent failure modes.	Baseline capacity does not predict recurrence, or apparent effects disappear after baseline burden and exposure are controlled.
P6	Randomized interface-cue experiment	Anthropomorphic confidence increases reliance on inaccurate outputs and accountability cues reduce it.	Reliance is unaffected, or cues improve reliance only for accurate outputs without increasing error acceptance.

P1 (individual; incremental validity): Among users matched on factual knowledge, health literacy, digital health literacy, AI literacy, and generalized misinformation resistance, a stronger pre-exposure GenAI-HIR configuration—represented as a domain profile or a composite only if the validated measurement model permits—will predict smaller confidence–accuracy error and safer action across mixed accurate and inaccurate high-stakes outputs.

P2 (cross-level moderation): Visible provenance, source dates, and calibrated uncertainty cues will strengthen the association between pre-exposure GenAI-HIR functions and independent verification behavior observed after exposure.

P3 (individual process): Pre-exposure interrogation competence will increase detection of missing red flags; red-flag detection will mediate its association with timely selection of qualified care, and pre-exposure escalation competence will strengthen the link between detection and action.

P4 (cross-level constraint): The protective association between individual functions and safe action will be weaker when authoritative verification or professional care is inaccessible, with greater attenuation among language, disability, and socioeconomic groups facing structural barriers.

P5 (organization; longitudinal): Baseline organizational source governance and incident-response capacity will predict fewer recurrent failure modes at follow-up, mediated by documented corrective-action completion rather than by a composite that already includes recurrence. A feasible design would recruit multiple sites using a common incident taxonomy and follow them across staged deployment or scheduled audit cycles.

P6 (interface experiment): Anthropomorphic and unwarrantedly confident interface cues will increase reliance on inaccurate outputs; explicit uncertainty and accountability cues will attenuate this direct effect, particularly among users with lower baseline recognition competence.

## 8 Candidate measures for future validation

### 8.1 Candidate individual and workforce indicators

Table 7 includes only 18 candidate self-report indicators for cognitive interviewing and item revision. They are not a measurement device. Prior to psychometric testing, each domain must also be illustrated with vignettes that are scored and consider source choice, omission detection, verification, contextual fit, and escalation. Cognitive interviews should determine whether statements refer to one ability or to a habit, belief or number of requirements, and should involve users that are diverse in language, age, disability, health literacy, and professional role. At this stage, no item, domain score, total score, threshold, certification or population comparison is justified.

**Table 7:** Candidate GenAI-HIR indicators for cognitive interviewing.

Domain	Item	Candidate statement
Recognize	R1	Given a health response, I can identify whether it was generated by AI or retrieved from a named professional source.
Recognize	R2	I can identify when an AI health answer concerns a high-stakes decision rather than general education.
Recognize	R3	I can identify when confident wording is not supported by source evidence.
Interrogate	I1	I can identify essential personal or clinical information missing from an AI health answer.
Interrogate	I2	I can ask a neutral follow-up question that tests an alternative explanation.
Interrogate	I3	I can identify which part of an AI answer depends on an unstated assumption.
Verify	V1	I can independently check a consequential AI health claim before acting on it.
Verify	V2	I can assess whether a cited source is authentic, current, and relevant to the same decision.
Verify	V3	I can explain why an AI system's self-critique is not independent verification.
Contextualize	C1	I can identify personal clinical factors that must be known before general guidance is applied.
Contextualize	C2	I can identify an access barrier that makes otherwise accurate guidance unusable.
Contextualize	C3	I can distinguish personalized wording from clinically valid individualization.
Escalate	E1	I can recognize when urgency or potential harm requires leaving the AI channel for qualified care.
Escalate	E2	I can select a proportionate level of care rather than treating every uncertainty as an emergency.
Escalate	E3	I can locate an accessible human-care route for the relevant setting.
Cross-capability	X1	I increase verification effort when potential harm is severe, urgent, or difficult to reverse.
Cross-capability	X2	I revise my judgment when independent evidence contradicts an AI answer.
Cross-capability	X3	After an unsafe answer, I can identify which step in the decision pathway failed.

### 8.2 Pilot descriptive reporting and organizational audit anchors

No missing data rule or domain summary rule is offered in the absence of cognitive and psychometric analyses. Early pilot reporting should be at the item level and include information on valid n, missingness, distributions of responses, comprehension issues, and vignette performance. The cross-capability indicators shall be kept separately. Only after dimensionality, reliability (if applicable), measurement invariance and criterion relationships support the relevant model, may domain or total scores be considered.

Table 8 lists descriptions of evidence within each domain in order, but is not a universal maturity ladder. Each reviewer should record the evidence and rationale for each domain separately; it is not appropriate to add the domains or average them or weight them or combine them into a single organizational rank. It is therefore possible for an organization to demonstrate standardized source control, and not demonstrate any tested human-routing pathway at the same time. The anchors are provided for content validation, gap identification, and repeat audit. For any maturity score, weighting scheme or threshold, there would be a need for separate empirical validation.

**Table 8:** Domain-specific organizational evidence anchors for content validation.

Domain	No documented control	Local or reactive evidence	Standardized evidence	Monitoring and adaptation evidence
Governance and use boundaries	No accountable owner or prohibited-use list	Local decisions without consistent documentation	Approved uses, owners, and review gates	Outcomes and incidents revise boundaries
Evidence/source control	No source standard	Manual checks vary by user	Curated sources, dates, jurisdiction, and audit logs	Drift and retrieval quality monitored by subgroup
Human escalation	Generic disclaimer only	Informal referral routes	Tested, role- and location-specific pathways	Completion, delay, and accessibility monitored
Equity/accessibility	No subgroup assessment	Reactive accommodation	Predeployment language, disability, and access testing	Subgroup outcomes trigger corrective action
Incident learning	No near-miss capture	Complaints handled individually	Taxonomy, root-cause review, and action ownership	Recurrence and time-to-correction monitored

### 8.3 Validation protocol

Future measurement work should proceed in stages (61). First, multidisciplinary experts and intended users should assess content coverage, clarity, redundancy, and accessibility through cognitive interviews. Second, behavioral vignettes should be developed alongside self-report indicators so perceived capability can be compared with observed decisions. Third, independent samples should be used to examine whether the functions behave as correlated reflective domains, a formative configuration, or a non-compensatory profile. Reliability and factor-fit indices are appropriate only for reflective models; formative evaluation should emphasize content coverage, collinearity, weight stability, and external prediction. Higher-order or total scoring should be considered only if the supported model permits it. Invariance must precede mean comparisons across languages, countries, age groups, professional groups, and disability groups. Criterion tests should use observed source choice, red-flag detection, verification, contextual adaptation, and proportionate escalation, with incremental validity assessed after established literacy measures.

## 9 Governance and implementation implications

### 9.1 Responsibility follows control

A distributed-responsibility model assigns each responsibility to the actor that can control the associated risk. Platforms manage interface cues, provenance displays, data use, model updates and use boundaries. Deploying organizations manage procurement, approved sources, workflow integration, easy escalation, incident management, monitoring, and corrective action. Authoritative control of information infrastructure, public guidance, surveillance and reporting pathways, and oversight are vested in public-health agencies. Community members and health communicators can tailor information, understand access challenges, and help people access services in their communities. Users and professionals use the functions possible through those conditions. All system vulnerabilities that are reasonably foreseeable should not be delegated to the least-resourced party via a blanket disclaimer (4–6, 43, 49, 55, 56, 59, 60, 62).

### 9.2 Risk-tiered deployment

Implementation should be rolled out from use limits to piloting, evaluation, scaling and adaptive management. Organizations should establish the outputs of the deployment, populations that will be impacted, prohibited use cases, accountable owners, approved sources, privacy controls, and validated escalation paths before deployment. Dangerous omissions, source accuracy, subgroup performance, escalation completion and correction time should be included in go-no-go criteria. Controls should reflect the level of the residual risk: a system that is acceptable for general education might not be appropriate for autonomous medication counseling or emergency triage.

### 9.3 Equity as constraint and outcome

Equity is not a function or part of an individual's score. Design constraint and outcome criterion. Assessments of languages/dialects, disability access, cultural expression, affordability assumptions, service availability, and working of escalation pathways should be done. Analyses should link differences between subgroups from exposure to action and outcome. Average accuracy does not equal equitable resilience when protection is based on resources that are not accessible to intended users (2, 51–59).

### 9.4 Public-health action for consumer tools outside organizational control

Public-health responsibility does not go away when people employ tools they have purchased or obtained without the involvement of a health organization or when they are used for reasons other than public health. Agencies can provide brief use limits, prebunk common mistakes, have multilingual verification pages and directories, track reported harms, and set up public reporting channels. Health communicators and community groups can modify those resources and identify routes that are inaccessible. Agencies can also ask for the platform-level provenance, safety-trigger and correction mechanisms, and procurement rules can be used to avoid uncontrolled outputs to formal workflows.

### 9.5 Worked example and resource-feasible implementation

Let's take the example of a health system that supplies a bilingual (English–Arabic) symptom-information chatbot. General education is supported, diagnosis is prohibited, autonomous triage is prohibited and medication changes are prohibited prior to launch. It relies on current national guidance, specifies the response language and offers human friendly routes. When the system is in use, a description of chest pain triggers a safety mechanism: it does not analyse the symptoms, it does not show any degree of certainty, it provides a route of urgency that is location specific and in the selected language and accessible format. Failure of routing, translation or completion goes into incident log – appropriate team corrects source/completes pathway – checks for recurrence by language and disability status. The Table 5 tier is connected to the same pathway as the actor duties in Figure 2 and the audit evidence in Table 8. It is recommended that resource-limited organizations adopt a minimum viable control set and not follow the most resource intensive model: reduce the supported use case, use a single verified source set, a single tested human path, and a small common set of incidents, and use shared national / regional verification services, if available. The number of scaling operations should be based on the risk observed and the capacity of the service. There is no requirement to provide an advanced “monitoring and adaptation” descriptor in order to provide information on any domain; it is evidence that the specific domain is currently being reviewed.

## 10 Research agenda

First, research needs to establish if the five functions constitute correlated domains, a formative configuration or a non-compensatory profile. Vignette experiments should manipulate accuracy, source quality, confidence, anthropomorphism, urgency, reversibility, and missing context, as well as assess confidence calibration, source selection, red-flag detection, verification time, and under- or over-escalation. Longitudinal studies need to be conducted in order to compare learning and over-reliance over multiple uses. Low-resource languages, disability groups and service systems should be incorporated in cross-cultural work. P5 should be used within organizational studies, and common incident definitions and staggered rollout or audit cycles should be used to make this feasible. Interrupted time-series or “natural experiments” may be used to evaluate platform or regulatory changes. Cost of source governance, accessible routing, monitoring and correction should be compared to costs of harm avoided, delayed services, unnecessary services and costs to restore trust.

## 11 Discussion

The main output of GenAI-HIR is a workable public-health governance model. The competency model is not validated when organizations use the output-risk procedure, actor responsibilities and domain-level audit anchors. The secondary contribution is a falsifiable research agenda: The proposed configuration should better predict calibration, verification, contextual adaptation or escalation over existing constructs of literacy. Otherwise, the five functions should continue to exist as an organizing checklist and not as a new psychometric construct. This separation helps overcome opposing forces of immediate application and subsequent construct validation.

The framework also identifies safe use and prompt proficiency. Whilst better prompts can enhance relevance, there can be a dis-juncture between fluent personalisation and clinical individualization. The aim is a pathway of decision in which individuals acknowledge the epistemic status and stakes, ask questions exposing gaps, explore consequences on their own, perceive contextual appropriateness, and act for human care if necessary. Organizational capacity provides the sources, routes, monitoring and accountability for those to function. Explanatory mechanisms and normative safeguards are also separated, avoiding the category error that would arise if risk mechanisms described what should be done to stop harm occurring, which is not the case.

### 11.1 Limitations

This is a conceptual synthesis based on theory, and includes no primary data, psychometric estimates, effect sizes, or causal identification. The candidate indicators, audit anchors, propositions and control tiers are preliminary. Purposive corpus is not comprehensive and may under-represent non-English scholarship, low-resource settings, disabled users, and reports of actual harm. Even if there is unanimous agreement on the meaning of the text, there can still be disagreement regarding its interpretation. Models, platforms and regulations evolve quickly and failure modes and potential controls will evolve as well. This framework is intended for use in conversational health-information and is not meant to be expanded to autonomous diagnostic devices and multimodal clinical systems without further analysis. Dimensionality, indicator performance, invariance, criterion relationships, incremental prediction, implementation cost and unintended effects must be tested in empirical work. Cautions should minimize over-reliance and should minimize the arbitrary dismissal of accurate and appropriately supported guidance.

## 12 Conclusion

Conversational GenAI is already part of the public-health information environment. GenAI-HIR offers a provisional governance structure for maintaining access to useful information while detecting, containing, correcting, and learning from consequential failures. Its five individual and workforce functions are conceptually distinguishable but not yet empirically separable; organizational capacity, platform conditions, and population outcomes remain analytically distinct. Immediate action is therefore governance-focused: public-health agencies should provide authoritative verification and prebunking; health systems should define use boundaries, source controls, accessible routing, and incident learning; platforms should expose provenance, uncertainty, reporting, and safety triggers; and educators should teach consequence-sensitive verification and escalation. The competency configuration should be treated as a testable research agenda until behavioral and psychometric evidence establishes whether it supports domain, profile, or composite interpretation.

## Appendix A Concept-development audit trail

The 62-reference list is the complete retained purposive corpus. Table A1 maps source clusters to analytic functions and decisions so readers can trace how the framework was constructed. Source-level judgments can be inspected using the stated coding fields: construct, level, mechanism, candidate function, decision, and justification.

**Table A1:** Retained-source clusters and decision contributions

Source cluster	References	Analytic function	Decision contribution
Public-health AI opportunities, harms, and equity	(1–3, 7–11, 18, 19, 45–47, 50–59)	Scope, mechanisms, and distributional consequences	Defined the focal exposure and separated equity as design constraint/outcome.
Health and digital health literacy	(24–29)	Adjacent constructs and existing measures	Supplied appraisal/application functions; showed need to test incremental rather than absolute novelty.
AI and algorithmic literacy	(30–33)	Model awareness and use competencies	Derived recognition; prevented reducing GenAI-HIR to prompt proficiency.
Inoculation and misinformation resistance	(17, 34–37)	Anticipatory resistance and correction	Derived recognition/prebunking while identifying excessive-skepticism negative case.
Epistemic vigilance	(38)	Source/message evaluation	Derived interrogation and independent verification logic.
Implementation and governance	(4–6, 20, 21, 39, 40, 43, 49, 55, 56, 60, 62)	Organizational embedding, oversight, and adaptation	Separated organizational capacity and enabling conditions from individual competencies.
LLM performance and human-automation evidence	(12–19, 41, 42, 44–50, 58)	Hallucination, fluency, advice acceptance, automation bias	Specified risk mechanisms and non-circular experimental outcomes.
Concept and scale methodology	(22, 23, 61)	Analytic method, cases, operationalization and validation	Anchored method; supplied model/borderline/contrary cases and staged validation plan.
